# Electrochemical Investigation of Curcumin–DNA Interaction by Using Hydroxyapatite Nanoparticles–Ionic Liquids Based Composite Electrodes

**DOI:** 10.3390/ma14154344

**Published:** 2021-08-03

**Authors:** Merve Uca, Ece Eksin, Yasemin Erac, Arzum Erdem

**Affiliations:** 1 Biotechnology Department, Graduate School of Natural and Applied Sciences, Ege University, 35100 Izmir, Turkey; merve.uca@hotmail.com; 2 Analytical Chemistry Department, Faculty of Pharmacy, Ege University, 35100 Izmir, Turkey; eceksin@hotmail.com; 3 Pharmacology Department, Faculty of Pharmacy, Ege University, 35100 Izmir, Turkey; yasemin.erac@ege.edu.tr

**Keywords:** hydroxyapatite nanoparticles, ionic liquid, bionanomaterials, nucleic acid interactions, curcumin, electrochemical biosensors

## Abstract

Hydroxyapatite nanoparticles (HaP) and ionic liquid (IL) modified pencil graphite electrodes (PGEs) are newly developed in this assay. Electrochemical impedance spectroscopy (EIS), scanning electron microscopy (SEM), energy-dispersive X-ray spectroscopy (EDX), and cyclic voltammetry (CV) were applied to examine the microscopic and electrochemical characterization of HaP and IL-modified biosensors. The interaction of curcumin with nucleic acids and polymerase chain reaction (PCR) samples was investigated by measuring the changes at the oxidation signals of both curcumin and guanine by differential pulse voltammetry (DPV) technique. The optimization of curcumin concentration, DNA concentration, and the interaction time was performed. The interaction of curcumin with PCR samples was also investigated by gel electrophoresis.

## 1. Introduction

In recent years, electrochemical nucleic acid biosensors have gained interest since they enable the observation of specific DNA hybridization and DNA interactions. These types of biosensors supply high sensitivity, simplicity, cost-effectiveness, and suitability for microfabrication [1,2,3]. In the field of electrochemical biosensors, nanoparticles have been widely used and they provide large surface area, high sensitivity, stability, and selectivity to electrochemical biosensors [4,5].

Hydroxyapatite (HaP), is one of the apatite mineral family [6] and is similar to the mineral constituent of bone. It is biocompatible, bioactive, and has particular multi-adsorbing sites [7]. Therefore, HaP is widely used in different areas such as the implementation of teeth and bone absorbents, separation of proteins, biosensors, and immunosensors [8]. Hap is mechanically stable, nontoxic, biodegrades slowly, and has excellent adsorption capacity [9]. Nanostructured HaP is more desired due to its higher surface area and strong adsorption ability [10,11]. However, there has been a limited number of hydroxyapatite-nanoparticles-modified electrochemical biosensors in the literature. Kanchana et al. [12] developed a novel biosensor using iron-doped hydroxyapatite nanoparticles and tyrosinase-modified glassy carbon electrodes. To detect l-tyrosine, amperometric methods were applied. In another study, a hydroxyapatite-based nonenzymatic biosensor was constructed for the determination of norepinephrine (NE), uric acid (UA), and tyrosine (Tyr) by differential pulse voltammetry (DPV) technique. The sensor was tested in human blood serum and urine samples [13]. Additionally, Erdem and Congur [7] developed hydroxyapatite-nanoparticles-modified pencil graphite electrode for the first time, and sequence-selective DNA hybridization was monitored by DPV technique.

Ionic liquids (ILs) have drawn attention and interest owing to their great features; for example, they are thermally stable, conductive, biocompatible, have low vapor pressure, low toxicity, and good solvation characteristics. At room temperature, they are found in a liquid phase and known as organic salts with low melting points [14,15]. The excellent properties of ILs allow for the development of biosensors in the field of biosensors [16,17], biomedicine [18], biocatalysis [19], and bioelectronics [20,21]. For instance, Sengiz et al. [14] demonstrated IL-modified pencil graphite electrodes (IL-PGEs) for monitoring DNA hybridization corresponding to *Microcystis spp.* (MYC) for the first time in the literature. Eksin et al. [22] developed chitosan and ionic liquid-based single-use PGEs (CHIT-IL-PGEs) to monitor the interaction of Mitomycin C (MC) with double-stranded DNA. The same group used CHIT-IL-PGEs to monitor the detection of sequence-selective DNA hybridization electrochemically [3]. In the manufacture of biosensors, ILs are used as an electrode modifier owing to their excellent ionic conductivity and wide electrochemical window [23,24,25]. Hence, ILs exhibit improved sensitivity of electrochemical DNA biosensors as a result of the enhanced electrochemical behavior of these electrodes [15].

Curcumin is a low-weight natural polyphenolic compound extracted from *Curcuma longa L.* [26,27]. Turmeric contains this phenolic pigment and curcumin is used in foods, drugs, and cosmetics to give color to colorless substances or change the color [28]. Curcumin has anti-inflammatory, antioxidant, chemotherapeutic, and cancer chemopreventive effect [29,30] The compound has presented a concentration dependency. As reported in the literature, no increase in the formation of reactive oxygen species (ROS) and damage in DNA was observed at low concentrations of curcumin [31]. On the other hand, no genotoxic effect was observed above 8 µg/mL concentration of curcumin [28]. The direct interaction of curcumin with nucleic acids has been approved by different studies. Generally, small molecules interact with DNA through three common binding ways, intercalation, groove binding, and electrostatic interaction [30].

The present study aimed to develop an electrochemical DNA biosensor that could be used in cancer. Curcumin is chosen as a DNA-targeted molecule since it is reported that the interaction of curcuminoids and their metal complexes with DNA occurs through groove binding [32,33,34,35,36,37,38] or intercalation [39,40]. There have been several methods applied for curcumin analysis such as UV–Vis spectrophotometry [41,42], liquid chromatography, [43], capillary electrophoresis [44], and mass spectrometry [45]. However, these techniques are time consuming, not portable, and have complex instrumentation [46]. In comparison to these techniques, electrochemical methods are cost effective, practical, require a short time, enable on-site analysis, and do not require toxic dyes such as ethidium bromide.

The aim of our work is to develop hydroxyapatite nanoparticles (HaP) and ionic liquid (IL)-based pencil graphite electrodes (HaP-IL-PGEs) as disposable, cost-effective, and sensitive DNA biosensor platforms to monitor curcumin–DNA interaction electrochemically. Under this aim, curcumin was chosen as the DNA-targeted molecule in this study. HaP-IL-PGEs were developed and applied for the first time in this study in a nucleic acid biosensor. The electrochemical and microscopic characterization of electrodes was studied by CV, SEM, EDX, and EIS techniques. The optimization of experimental parameters, curcumin concentration, DNA concentration, and the interaction time of curcumin with DNA was studied by using the differential pulse voltammetry (DPV) technique. Under the optimum conditions, the interaction of curcumin with ctDNA was investigated. We aimed to develop an electrochemical DNA biosensor that could be used in cancer treatment research studies. To the best of our knowledge, the voltammetric detection of interaction of curcumin with ctDNA was reported for the first time herein by using hydroxyapatite nanoparticles and ionic liquid-modified pencil graphite electrodes (HaP-IL-PGEs). Since HaP and IL modification onto biosensor surface provides a greater surface area, it was applied herein for amplified detection of curcumin and also nucleic acids. Moreover, the interaction of curcumin with the PCR samples, which were obtained by isolating cDNA from Huh7 human hepatocellular carcinoma cell line by polymerase chain reaction (PCR), was monitored by gel electrophoresis technique as a reference method for comparison to the results obtained by DPV technique.

## 2. Materials and Methods

### 2.1. Instruments

AUTOLAB PGSTAT 302 with an FRA 2.0 module and μAUTOLAB PGSTAT-204 with NOVA 1.11 software (2014, Version 1.11.0, Eco Chemie, Uthrecht, The Netherlands) were used for impedimetric measurements and voltammetric measurements, respectively. Pencil graphite electrode (PGE, Tombow 0.5 HB, Tokyo, Japan), an Ag/AgCl/3 M KCl, and a platinum wire were used as the working, reference, and counter electrode, respectively, which constitute the three-electrode system. A Rotring 0.5 mechanical pencil (Rotring, Hamburg, Germany) was used in order to hold the electrode. Electrical conductivity was provided with copper wire. About 1 cm of the pencil graphite was immersed into 2 mL of analysis solution in an electrochemical cell. EIS measurements were performed in Faraday cage (Eco Chemie, Uthrect, The Netherlands).

### 2.2. Chemicals

Hydroxyapatite nanoparticles (HaP) solution, the ionic liquid (IL), 1-butyl-3 methylimidazolium hexafluorophosphate, double-stranded calf thymus DNA (ctDNA), and curcumin were purchased from Sigma-Aldrich (St. Louis, USA). The stock solution of ctDNA was prepared as 1000 µg/mL in Tris-EDTA buffer solution (pH = 8.00) and kept frozen. Diluted solutions of ctDNA were prepared by using 0.05 M acetate buffer containing 20 mM NaCl (ABS, pH 4.8).

Other chemicals were in analytical reagent grade, and they were supplied from Sigma Aldrich (St. Louis, MI, USA) and Merck (Darmstadt, Germany). Ultrapure water was used in all solutions.

### 2.3. Procedure

The whole procedure was carried out at room temperature. Each graphite electrode had a pretreatment step in which +1.40 V was applied during 30 s in acetate buffer solution (ABS, pH, 4.8).

The experimental procedure steps are as follows:

(i) Electrochemical pretreatment of PGEs;
(ii) Surface modification of PGE with HaP and IL, respectively;
(iii) ctDNA (calf thymus double-stranded DNA) immobilization onto the surface of HaP-IL-PGE;
(iv) Electrochemical detection of curcumin–ctDNA interaction by HaP-IL-PGE.

The mentioned procedure is presented in Scheme S1 given in Supplementary Materials.

#### 2.3.1. Preparation of HaP-PGEs

N,N-dimethylformamide (DMF) was used to dilute the stock solution of HaP (10^5^ µg/mL). A total of 100 µg/mL of HaP was dissolved in DMF by sonication for 15 min. Each pretreated pencil lead was dipped the required amount of HaP for 30 min. Then, electrodes were washed with ABS. Next, the HaP-PGEs were dried for 5 min at an upside-down position.

#### 2.3.2. Preparation of HaP-IL-PGE

N,N-dimethylformamide (DMF) was used to dilute the 5% IL, and then sonication was applied for 30 min at room temperature. Each of the HaP-PGEs was dipped into 5% ionic liquid for 1 h, as explained in previous studies [14,22]. Next, HaP-IL-PGEs were dried for 20 min at an upside position without any washing step.

#### 2.3.3. Curcumin Immobilization on HaP-IL-PGEs

HaP-IL-PGEs were dipped into 10 µg/mL of curcumin solution for 5 min at an upside-down position. Immobilization of curcumin onto the electrode surfaces was carried out with wet adsorption in dark due to the photolytic instability of curcumin [47]. Next, the electrodes were washed with ABS to eliminate unbound curcumin from the electrode surface.

#### 2.3.4. ctDNA Immobilization on HaP-IL-PGEs

HaP-IL-PGEs were dipped into the 25µg/mL of ctDNA in ABS for 1 h. Next, the electrodes were washed with ABS to eliminate unbound DNA from the electrode surface.

#### 2.3.5. Interaction of Curcumin with ctDNA at the Surface of HaP-IL-PGEs

ctDNA immobilized HaP-IL-PGEs were dipped into the 10 µq/mL of curcumin, and interaction was allowed for 3 min. Next, the electrodes were washed with ABS before voltammetric measurement.

#### 2.3.6. Preparation of PCR Samples

Isolation of total RNA from Huh7 human hepatocellular carcinoma (HCC) cell line was performed by using UltraClean^®^ Tissue and Cells RNA Isolation Kit (MO BIO Laboratories, Inc., Carlsbad, CA, USA) according to the manufacturer’s instructions. Human HCC cell lines (Huh-7) were provided by Dr. Ozturk (Izmir Biomedicine and Genome Center, Izmir, Turkey), originally from Dr. Jack Wands Laboratory (Massachusetts General Hospital, Boston, MA, USA), as a gift. Determination of RNA concentration was studied by measuring the absorbance at 260 nm in a spectrophotometer (Nanovete, Beckman Coulter, Brea, USA). cDNA synthesis was performed with EasyScript Plus™ cDNA Synthesis Kit (Lamda Biotech, St. Louis, MO, USA) using random primers. Polymerase chain reaction (PCR) was accomplished using PCR-EZ D-PCR Master Mix (Bio Basic Inc., St. Louis, MI, USA) and thermal cycler (Techne, ThermoFisher, Waltham, MA, USA).

The 18S ribosomal 5 (RNA 18S5) primer sequences used in PCR are 5′- CGA CGA CCC ATT CGA ACG TCT-3′ and 5′-G CTA TTG GAG CTG GAA TTA CCG-3′ to generate 312 bp product. GC content of PCR product is 54%.

### 2.4. Electrochemical Measurements

DPV measurements were performed in ABS (pH, 4.8) by scanning from +0.2 V to +1.45 V at a pulse amplitude of 50 mV and a scan rate of 50 mV/s. CV measurements were performed with the following parameters: a step potential of 25 mV/s; a scan rate of 50 mV/s; forward scan, −0.8 to +1.4 V; reverse scan, +1.4 to −0.8 V.

### 2.5. Impedimetric Measurements

A total of 2.5 mM K_3_[Fe(CN)_6_]/K_4_[Fe(CN)_6_] (1:1) in 0.1 M KCl was used as a redox probe solution for impedimetric measurements. Measurements were carried out with the frequency range from 100 mHz to 100 kHz in an open circuit potential of +0.24 V, with a pulse amplitude of 10 mV, and in a Faraday cage. The charge transfer resistance (R_ct_) represents the respective semicircle diameter, and calculations were performed by the fitting program AUTOLAB 302 with an FRA 2.0 module.

### 2.6. Microscopic Characterization

Quanta 400 FEI, scanning electron microscope (FEI Company, Tokyo, Japan) was used for acquiring the surface morphologies of modified and unmodified electrodes. Images obtained at different acceleration voltages as 5.0 kV and 7.0 kV and the 10 µm and 3 µm resolutions were used.

### 2.7. Gel Electrophoresis

Preparation of non-denaturing agarose (2%) gels was performed in 1X TAE buffer. (1) PCR, (2) curcumin, and (3) curcumin-treated PCR samples (incubated with curcumin for 1, 3, and 5 min) were loaded into the wells with gel loading dye (6X concentration, 0.25 bromphenol blue, 0.25% xylene cyanol, 30% glycerol). TAE buffer was used for electrophoresis for 30 min at 100 V. After agarose gel electrophoresis, the gel was spotted with ethidium bromide (EtBr, 2%), and visualization was conducted by a transilluminator (Vilber Lourmat, Marne La Vallee, France).

## 3. Results and Discussion

**Figure 1:**
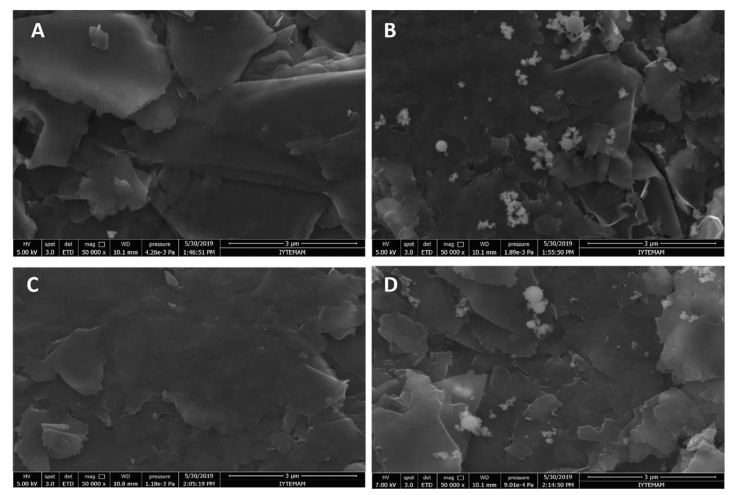
SEM images of the PGEs (**A**), HaP-PGEs (**B**), IL-PGEs (**C**) using identical acceleration voltage 5.0 kV, and HaP-IL-PGEs (**D**) using identical acceleration voltage 7.0 kV with the resolution at 3 µm, respectively.

First, the surface properties of the PGE, HaP-PGE, IL-PGE, and Hap-IL-PGE were examined by SEM, and the images are given in Figure 1. The layered graphite structure of PGEs was observed, as shown in Figure 1A. After HaP modification, the nanoparticles were detected on the surface of PGEs (Figure 1B). IL modification onto PGE makes PGE surface smoother, leading to the coating of conductive IL compound (Figure 1C) [15,16]. As can be seen in Figure 1D, the presence of hydroxyapatite nanoparticles and the smooth surfaces supply information about the successful coating of PGE with HaP-IL.

The results of the EDX analysis are shown in Figure S1. The elemental compositions of each electrode PGE, HaP-PGE, IL-PGE and HaP-IL-PGE are indicated as A, B, C, and D, respectively. According to the EDX result of HaP-PGE (shown in Figure S1B), the Ca/P atomic ratio of the HaP is calculated as 1.98, which is close to the stoichiometric ratio of HaP (Ca/P = 1.67) reported in the study of Rassaei et al. [4]. In an assay, results showed that sintered hydroxyapatite powder at 900 °C whose Ca/P ratio varies 1.7 between 2.4 showed the properties of pure and single-phase apatite form [48]. It can be concluded that the modification of hydroxyapatite nanoparticles onto the surface was performed successfully. Figure S1C illustrates the elemental composition of IL mainly based on phosphorus and fluorine that is also similarly reported in the study of Hernández-Fernández et al. [49]. All elements existing in the structure of HaP and IL can be observed in the EDX spectrum of HaP-IL-modified PGE (Figure S1D). As a result, it was found that the results of EDX and SEM were in good agreement.

**Figure 2:**
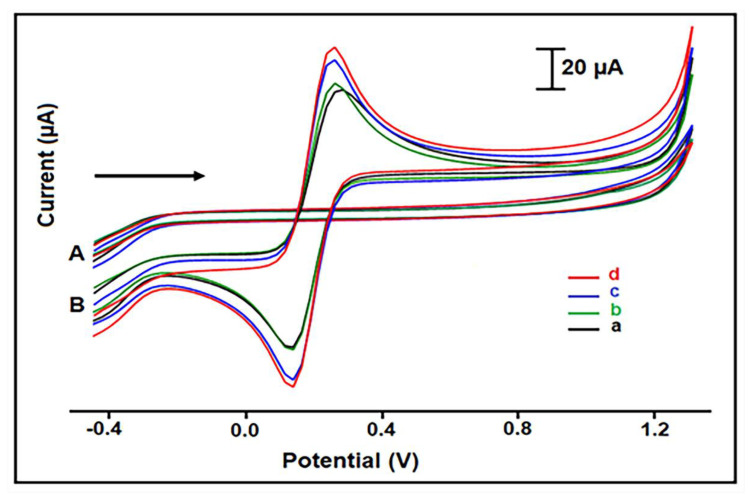
CVs obtained by (**a**) PGE, (**b**) HaP-PGE, (**c**) IL-PGE, and (**d**) HaP-IL-PGE. Measurements were performed in 0.1 M KCl (**A**), in ferri/ferro redox system prepared in 0.1 M KCl (**B**).

CV measurements of PGE, HaP-PGE, IL-PGE, and HaP-IL-PGE were performed in 2 mM K_4_[Fe(CN)_6_]/K_3_[Fe(CN)_6_] (1:1) containing 0.1 M KCl to investigate the electrochemical performance of each electrode (Figure 2B). Symmetrically and well-defined peak shapes were obtained at +0.26 V (anodic peak, E_pa_) and +0.13 V (cathodic peak, E_pc_). The anodic peak current (I_a_) of HaP-IL-PGE (Figure 2d) was larger than I_a_ of IL-PGE (Figure 2c), HaP-PGE (Figure 2b), and PGE (Figure 2a). Cathodic peak current (I_c_) shows the same behavior.The average I_a_ values for electrodes are given in Table S1. The conductivity of IL and the ability of HaP to enable high surface area [7,50,51,52,53] provided an enhancement at the I_a_ signal of HaP-IL-modified PGE, compared to signals of PGE, HaP-PGE, and IL-PGE. Additionally, CV measurements were performed in 0.1 M KCl without K_4_[Fe(CN)_6_]/K_3_[Fe(CN)_6_] redox system as a control experiment, and there was no signal, as shown in Figure 2A.

The effective surface area (*A_eff_*) of electrodes was calculated by using the Randles–Sevcik equation [54], and they are all given in Table S1. The largest surface area was obtained with HaP-IL-modified PGE as 0.35 cm^2^ due to the fact that HaP modification increases the surface area, and also, more electrons can transfer. Moreover, IL increases the conductivity that results in an increase in current. Hence, this surface modification increases the surface area for more binding of DNA onto the electrode surface.

**Figure 3:**
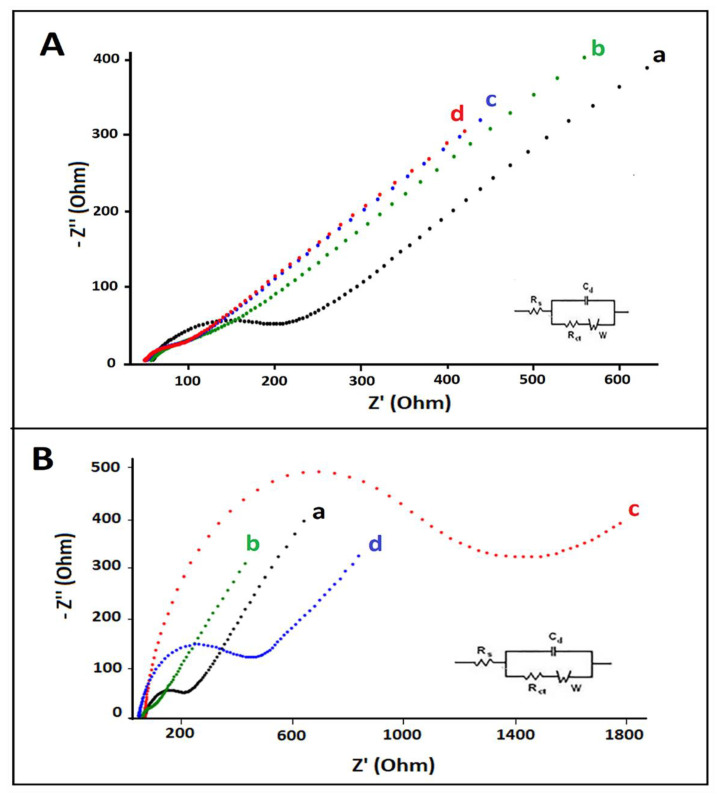
(**A**) Nyquist diagrams of (a) PGE, (b) HaP-PGE, (c) IL-PGE, and (d) HaP-IL-PGE; (**B**) Nyquist diagrams of (a) PGE, (b) HaP-IL-PGE, (c) ctDNA immobilized PGE, (d) ctDNA immobilized HaP-IL-PGE. An equivalent circuit model was used to obtain impedance data shown in inset, where C_d_ is the constant phase element associated with the space charge capacitance, the solution resistance showed as R_S_ and R_ct_ involved with the resistance of charge transfer at the DNA/electrolyte interface, and W is the Warburg impedance.

The EIS technique was also used as another surface characterization technique. The real and imaginary impedance (Z’ and −Z”) are the components of the complex impedance (Z) (Figure 3A,B). An equivalent circuit model (Randles circuit) was utilized for fitting impedimetric results (Figure 3A,B inset). According to Randles circuit, R_s_ is the solution resistance. C_d_ is the double-layer capacitance. The charge transfer resistance (R_ct_) is defined as the resistance related to the dielectric and insulating characteristics at the electrode/electrolyte interface. W is the Warburg impedance due to mass transfer to the electrode surface and observed at higher frequencies [8,23]. The average R_ct_ of electrodes with the decrease% at R_ct_ values in comparison to PGE is given in Table S2, and Nyquist diagrams are shown in Figure 3A. In contrast to the R_ct_ value measured by PGE, lower R_ct_ values were obtained by using HaP-PGE, IL-PGE, and HaP-IL-PGE. This decrease could be attributed that the ability of HaP to increase the surface area [7], while IL is increasing the conductivity [3].

Following 25 µg/mL ctDNA immobilization on PGE, R_ct_ value was recorded as 1120.0 ± 99 Ohm (RSD% = 8.8%, *n* = 3) with 9.6-fold increase, compared to PGE. The R_ct_ value of ctDNA immobilized HaP-IL-PGE was recorded as 304.5 ± 14.9 Ohm (RSD% = 4.9%, *n* = 3), with 9.3-fold increase, compared to HaP-IL-PGE (Figure 3B). The repulsion between the phosphate backbone of ctDNA and anionic redox probe [Fe(CN)_6_]^4−/3−^ increased the R_ct_ values [55]. Additionally, the calculation of fractional coverage values (Q_ıs_^R^) was performed for 25 µg/mL ctDNA immobilized on PGE and HaP-IL-PGE according to Equation (1) given by Janek et al. [56] and calculated as 0.89 and 0.90, respectively. Successful coating of the surface of PGE resulted in a higher surface area to bind ctDNA, which gives a higher fractional coverage value with HaP-IL-modified PGE.

(1) $$Q_{IS}^{R}=1-\frac{R_{ct}^{HaP-IL-PGE/\;PGE}}{R_{ct}^{ctDNA\;HaP-IL-PGE/\;PGE}}\;\;$$

Cyclic voltammetry technique was performed to optimize the HaP concentration in case of constant IL concentration as 5%. The changes at the anodic peak (I_a_) values of -Fe(CN)_6_^3−/4−^ were estimated in the sense of modification procedure. The highest increases 21.6% and 6.8% were obtained in the presence of 100 µg/mL HaP solution, and I_a_ was recorded as 98.4 ± 11.2 µA (RSD% = 11.4%, *n* = 13) and 120.5 ± 5.9 µA (RSD% = 4.9%, *n* = 13) by using PGE and 5% IL-PGE, respectively (Figure S2).

In order to illustrate how IL concentration affects the sensor response, different IL concentrations of 2.5%, 5%, and 10% were examined, and results are given in Figure S3. The average peak currents (I_a_) measured in the absence and presence of 100 µg/mL HaP are given in Table S3. The highest peak currents were recorded in the presence of 5% IL at both PGE and HaP-PGE, with the average signals as 108.7 ± 7.0 µA (RSD% = 6.4%, *n* = 9) and 115.9 ± 8.7 µA (RSD% = 7.5%, *n* = 9), respectively. Thus, the highest increase in signals was evaluated as 24.3% and 29.4% by using PGE and HaP-PGE, respectively (Figure S3).

**Figure 4:**
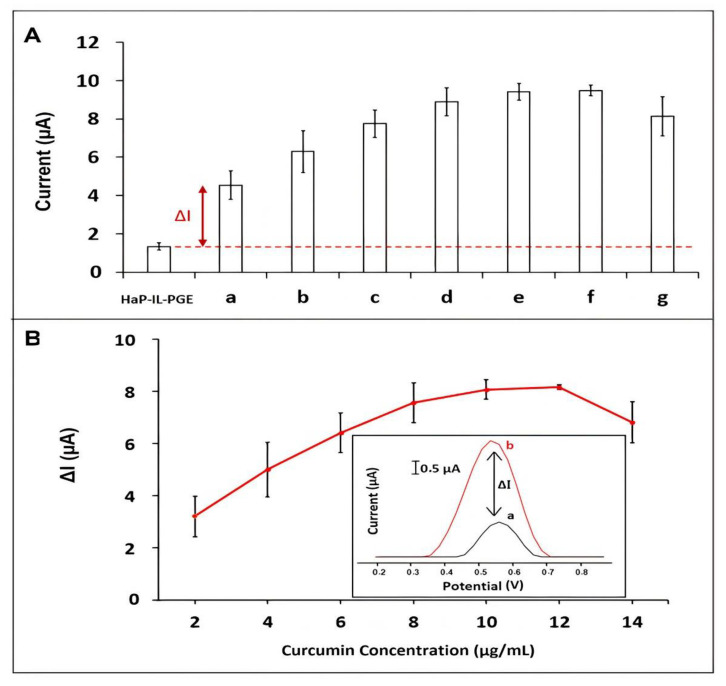
(**A**) Histograms related to the average curcumin oxidation signals (*n* = 9) recorded in the presence of (a) 2, (b) 4, (c) 6, (d) 8, (e) 10, (f) 12 and (g) 14 µg/mL curcumin onto the HaP-IL-PGEs; (**B**) the line graphs illustrate the curcumin oxidation signal obtained in the presence of various Curcumin concentrations from 2 to 14 µg/mL by using HaP-IL-PGE. Inset: (a) signal measured in ABS buffer in control experiment at + 0.56 V peak potential by HaP-IL-PGE; (b) signal measured in ABS buffer at + 0.56 V peak potential after curcumin immobilization.

To optimize the curcumin concentration, the changes at curcumin oxidation signals were examined by HaP-IL-PGE ranging from 2 to 14 µg/mL with the DPV technique. After the concentration of 10 µg/mL curcumin, signals stayed constant and started to decrease. The optimum curcumin concentration was chosen as 10 µg/mL since the highest oxidation signal (8.1 ± 0.4 µA (RSD% = 4.6%, *n*=9)) and the highest increase (2.10 fold) obtained in comparison to PGE (Figure 4A,B). All calculations were carried out based on the equation ΔI= b-a since the peak potential of HaP-IL-PGE was overlapped with the oxidation signal of curcumin at the same peak potential (Figure 4B). In the equation, “b” implies the oxidation signal measured at +0.56 V peak potential after curcumin immobilization onto the surface HaP-IL-PGE, and “a” implies the oxidation signal measured at +0.56 V peak potential in the absence of curcumin by HaP-IL-PGE.

The detection limit [57] of curcumin was calculated by the regression equation y = 0.62 x + 2.36 and R^2^ = 0.96 for HaP-IL-PGE (Figure S4) and found to be 1.86 µg/mL (equals to 5.04 µM) by HaP-IL-PGE, with the sensitivity of 1.76 µA mL µg^−1^ cm^−2^. Therefore, it can be concluded that the sensitive curcumin analysis could be performed at lower concentration value of curcumin than 2 µg/mL by using HaP-IL-PGE.

To examine how the changes in DNA concentration affect the response, a batch of the experiment was performed in the presence of different ctDNA concentration levels varying between 5 to 30 µg/mL. The guanine oxidation signal was measured at +1.0 V potential by the DPV technique. The highest guanine oxidation signal was recorded as 6.1 ± 0.2 µA (RSD% = 3.4%, *n*=3) after the immobilization of 25 µg/mL ctDNA onto the surface of HaP-IL-PGE (Figure S5). Hence, 25 µg/mL was chosen as optimum ctDNA concentration, with a 2.05-fold increase in comparison to PGE.

The detection limit [57] of ctDNA was calculated in the linear range varying 5–30 µg/mL by using the regression equation y = 0.22 x + 0.90, R^2^ = 0.99 and found to be 2.42 µg/mL by HaP-IL-PGE (Figure S6), with the sensitivity of 0.61 µA mL µg^−1^ cm^−2^.

**Figure 5:**
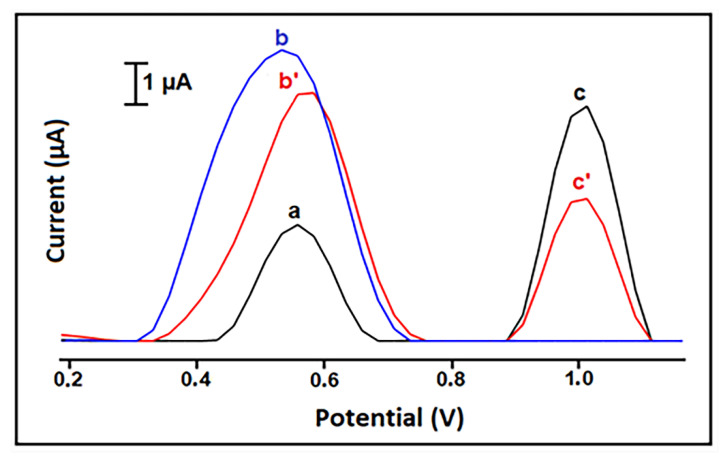
Voltammograms representing the oxidation signal of 10 µg/mL curcumin and the oxidation signal (i.e., guanine signal) of 25 µg/mL ctDNA measured before and after 3 min interaction: (a) the control signal measured by HaP-IL-PGE, oxidation signal of curcumin (b) before, (b’) after interaction, oxidation signal of guanine (c) before, (c’) after interaction.

Next, the interaction between curcumin and ctDNA was analyzed at the surface of HaP-IL-PGE with DPV. The surface-confined interaction between 10 µg/mL curcumin and 25 µg/mL DNA was investigated in different interaction times; 1, 3, and 5 min. The changes at the curcumin and guanine oxidation signals before and after interaction are given in Table S4 and Table 1, respectively. The highest decrease ratio % (20.2%) at the curcumin oxidation signal was recorded after 3 min interaction (Table S4). The highest decrease (43.2%) in the guanine signal was obtained after 5 min interaction. However, in the case of 3 min interaction, more reproducible signals were obtained, in contrast to the signals obtained after 5 min interaction (Table 1); optimum interaction time was selected as 3 min. The representative voltammograms for this part are shown in Figure 5.

**Figure 6:**
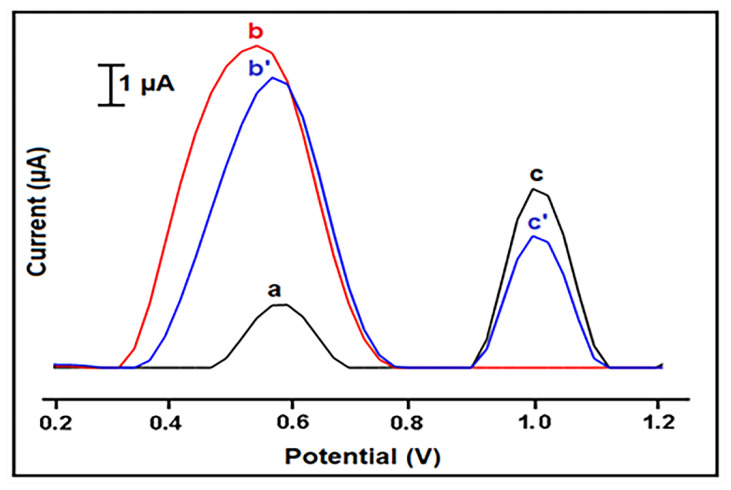
Voltammograms representing the oxidation signal of 10 µg/mL curcumin and the oxidation signal (i.e., guanine signal) of 25 µg/mL PCR sample measured before and after 3 min interaction: (a) the control signal measured by HaP-IL-PGE, oxidation signal of curcumin (b) before, (b’) after interaction, oxidation signal of guanine (c) before, and (c’) after interaction.

Additionally, the interaction between 10 µg/mL curcumin and 25 µg/mL PCR sample was studied at different interaction times. The oxidation signal of curcumin and guanine signals were measured before and after the 1, 3, and 5 min interaction process. Accordingly, the calculations were performed based on the changes at the signals presented in Tables S5 and S6. The most decrease in both curcumin and guanine oxidation signal was recorded with 3 min interaction process. Figure 6 shows the representative voltammograms. As a result, it can be concluded that the results were compatible with the one obtained by ctDNA.

**Figure 7:**
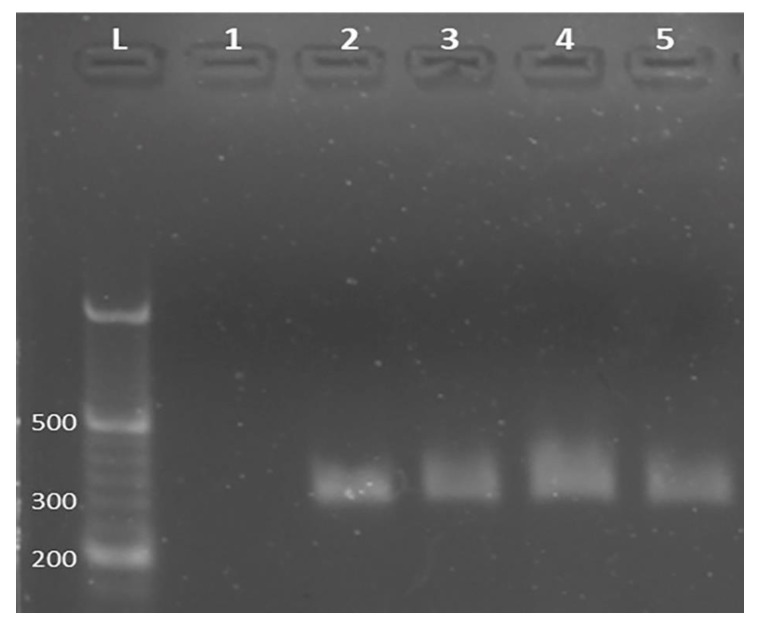
Non-denaturing agarose (2%) gel electrophoresis of 10 µg/mL curcumin (lane 1), 25 µg/mL PCR sample in the absence of curcumin (312 bp, lane 2), and 10 µg/mL curcumin-incubated PCR samples (25 µg/mL) during 1, 3, and 5 min (lane 3, 4, and 5, respectively). In all incubated PCR samples with curcumin, the ethidium bromide intensity decreased, compared to control PCR sample (lane 2). L: 50 bp DNA ladder.

Confirmation of the interaction between curcumin and PCR was ensured by using gel electrophoresis. Agarose gel electrophoresis has been commonly used to determine DNA structural change [57,58,59,60,61]. To confirm electrochemical experiment results, the control agarose gel electrophoresis experiment was performed to determine DNA damage caused by curcumin incubation. As a result of curcumin–DNA incubation during 1, 3, and 5 min, ethidium bromide intensity was decreased, in comparison with the presence of only PCR sample (Figure 7). The decrease in ethidium bromide intensity may be a consequence of the interaction of curcumin with DNA. Furthermore, the DNA sample that was incubated with curcumin for 3 min presented mildly longer migration, compared to control and other DNA samples. In conclusion, curcumin might be responsible for the change in DNA secondary structure. As a result, one may conclude that, under these conditions, transition in confirmation of DNA occurred during incubation with TD, which can be attributed to the modification of the DNA secondary structure [57,58,59,60,61,62].

## 4. Conclusions

Hydroxyapatite nanoparticles (HaP)- and ionic liquid (IL) modified electrochemical DNA biosensor was introduced for the first time in the present study. It was aimed to develop an electrochemical DNA biosensor that could be developed furtherly for cancer research studies. Curcumin was chosen as the DNA-targeted molecule since it is known that the interaction of curcuminoids and their metal complexes with DNA occurs through groove binding or intercalation. The detection limit of curcumin was calculated and found to be 1.86 µg/mL (equals to 5.04 µM) by HaP-IL modified electrodes. Curcumin has both genotoxic and antigenotoxic effects. Above 8 µg/mL, it increases the formation of reactive oxygen species, which causes damage in DNA [28]. The surface confined interaction of curcumin with ctDNA and PCR samples isolated from Huh7 human hepatocellular carcinoma cell line was detected voltammetrically. According to the changes observed at at the oxidation signals of curcumin and guanine in various interaction times, the optimum interaction time of curcumin with ctDNA was chosen as 3 min. Moreover, the results of interaction of curcumin–ctDNA and curcumin–PCR samples were found to be in good agreement with each other. Additionally, the interaction of curcumin with PCR samples was studied with agarose gel electrophoresis as the reference method. The consistency in the voltammetric results and the gel electrophoresis results were observed. HaP-IL-PGEs proved to be cost-effective and sensitive DNA biosensor platforms to monitor electrochemically DNA and its interactions with curcumin.

This assay, in combination with the HaP-IL-modified disposable biosensor, can be used for sensing biomolecules such as nucleic acids, proteins, and drugs. In conclusion, this type of biosensor-based assay could elicit the development of novel biorecognition tools to understand the effect of biomolecules such as curcumin on genetic material, e.g., nucleic acids.

## Figures and Tables

**Table 1:** The average guanine signals in case of different interaction times and the decrease% ratios after interaction of curcumin–ctDNA (*n* = 6).

Interaction Time (min)	Guanine Signal before Interaction (µA)	Guanine Signal after Interaction (µA)	Decrease % at Guanine Signal
1	6 ± 0.9 (RSD% = 14.7%)	4.3 ± 0.5 (RSD% = 10.9%)	29.3%
3		3.9 ± 0.3 (RSD% = 8.2%)	36.3%
5		3.4 ± 0.6 (RSD% = 16.7%)	43.2%

## Data Availability

The data presented in this study are available within the article and its Supplementary Materials. Other data that support the findings of this study are available upon request from the corresponding author.

## Supplementary Materials

The following are available online at https://www.mdpi.com/article/10.3390/ma14154344/s1, Scheme S1: The schematic model for the construction of HaP-IL-PGE, immobilization of DNA or Curcumin, and the electrochemical monitoring of Curcumin-ctDNA interaction with HaP-IL-PGEs; Figure S1: Graphs representing the elemental percentages of (**A**) PGE, (**B**) HaP, (**C**) IL and (**D**) HaP-IL-PGE obtained by EDX analysis; Figure S2: Histograms representing the average oxidation signals measured before and after modification of different HaP concentrations onto the electrode surfaces in the presence of 5% IL ( *n* = 13); Figure S3: Histograms representing the average oxidation signals measured before and after modification of different IL percentages onto the electrode surfaces in the presence of 100 µg/mL HaP (*n* = 9); Figure S4: Calibration plot based on the average Curcumin oxidation signals in the presence of various Curcumin concentrations between 2 and 10 µg/mL using HaP-IL-PGEs (*n* = 9); Figure S5: (**A**) DPVs representing the average guanine signals obtained after immobilization of (a) 5, (b) 10, (c) 15, (d) 20, (e) 25, (f) 30 µg/mL ctDNA onto the surface of HaP-IL-PGEs. (**B**) The line graph based on the average guanine oxidation signals obtained by HaP-IL-PGE (*n* = 3); Figure S6: Calibration plot based on the average guanine oxidation signals in the presence of various DNA concentrations between 5 and 25 µg/mL using HaP-IL-PGEs (*n* = 3); Table S1: The average anodic peak currents (Ia) with their calculated surface areas (*n* = 3); Table S2: The average Rct values of each of electrodes (*n* = 3) with the decrease % ratio calculated contrast to the one of PGE; Table S3:The effect of IL % onto the response with the average anodic peak currents (Ia) (*n* = 9); Table S4: The average oxidation signals of Curcumin before and after different interaction times and change ratios after interaction process between Curcumin and ctDNA (*n* = 6); Table S5: The average oxidation signals of Curcumin before and after different interaction times and decrease ratios after interaction process between Curcumin and PCR samples (*n* = 6); Table S6: The average oxidation signals of guanine before and after different interaction times and change ratios after interaction process between Curcumin and PCR samples (*n* = 6).
