# Supplementary material for: Electrochemical Investigation of Curcumin–DNA Interaction by Using Hydroxyapatite Nanoparticles–Ionic Liquids Based Composite Electrodes
Source: Materials (Basel). 2021 Aug 3;14(15):4344. doi: 10.3390/ma14154344 (PMC8347690; doi:10.3390/ma14154344)
Supplement: Supplementary file 1 [file materials-14-04344-s001.zip › materials-1280029 - supplementary.pdf]

materials

Supplementary Materials

Electrochemical Investigation of Curcumin–DNA Interaction by Using Hydroxyapatite Nanoparticles–Ionic Liquids Based Composite Electrodes

Merve Uca [1,2], Ece Eksin [2], Yasemin Erac [3] and Arzum Erdem [1,2,*]

[1] Biotechnology Department, Graduate School of Natural and Applied Sciences, Ege University, 35100 Izmir, Turkey; merve.uca@hotmail.com
[2] Analytical Chemistry Department, Faculty of Pharmacy, Ege University, 35100 Izmir, Turkey; eceksin@hotmail.com
[3] Pharmacology Department, Faculty of Pharmacy, Ege University, 35100 Izmir, Turkey; yasemin.erac@ege.edu.tr
* Correspondence: arzum.erdem@ege.edu.tr or arzume@hotmail.com

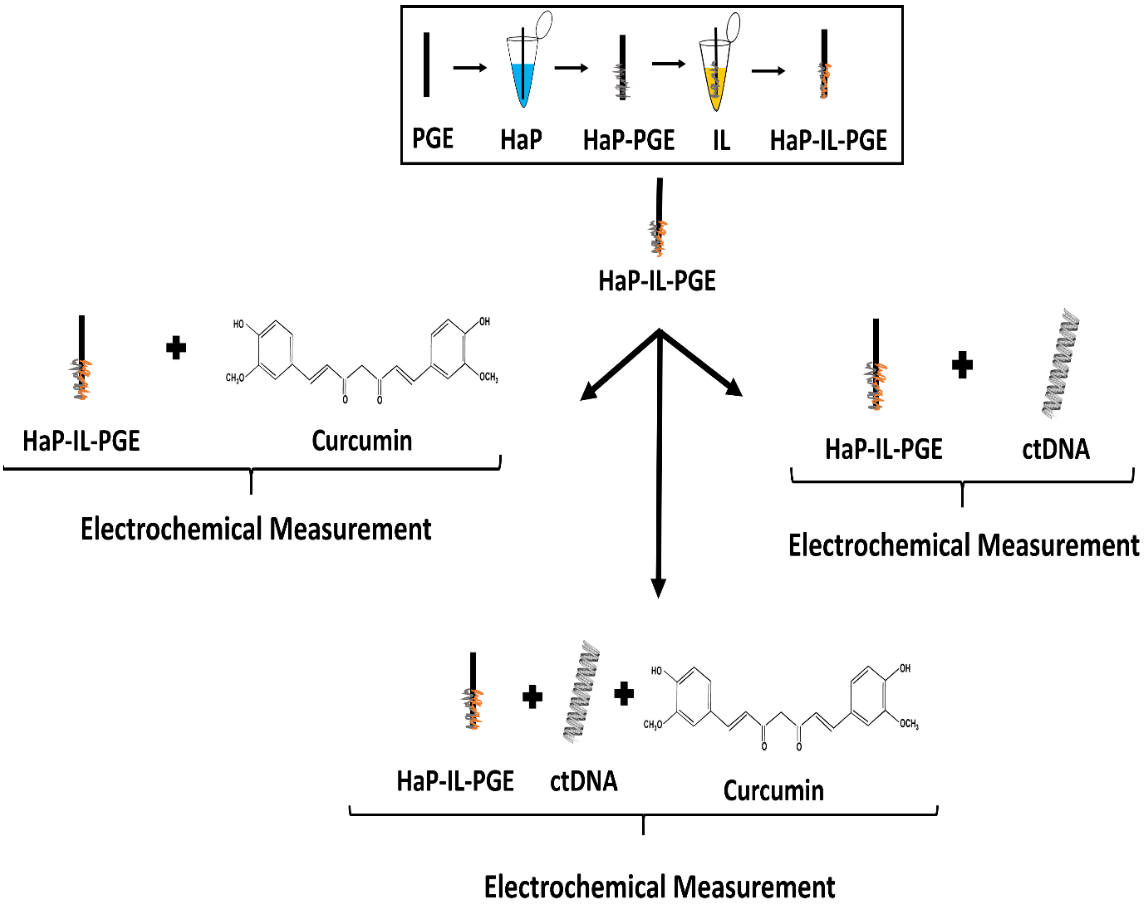

Scheme S1. The schematic model for the construction of HaP-IL-PGE, immobilization of DNA or Curcumin, and the electrochemical monitoring of Curcumin-ctDNA interaction with HaP-IL-PGEs.

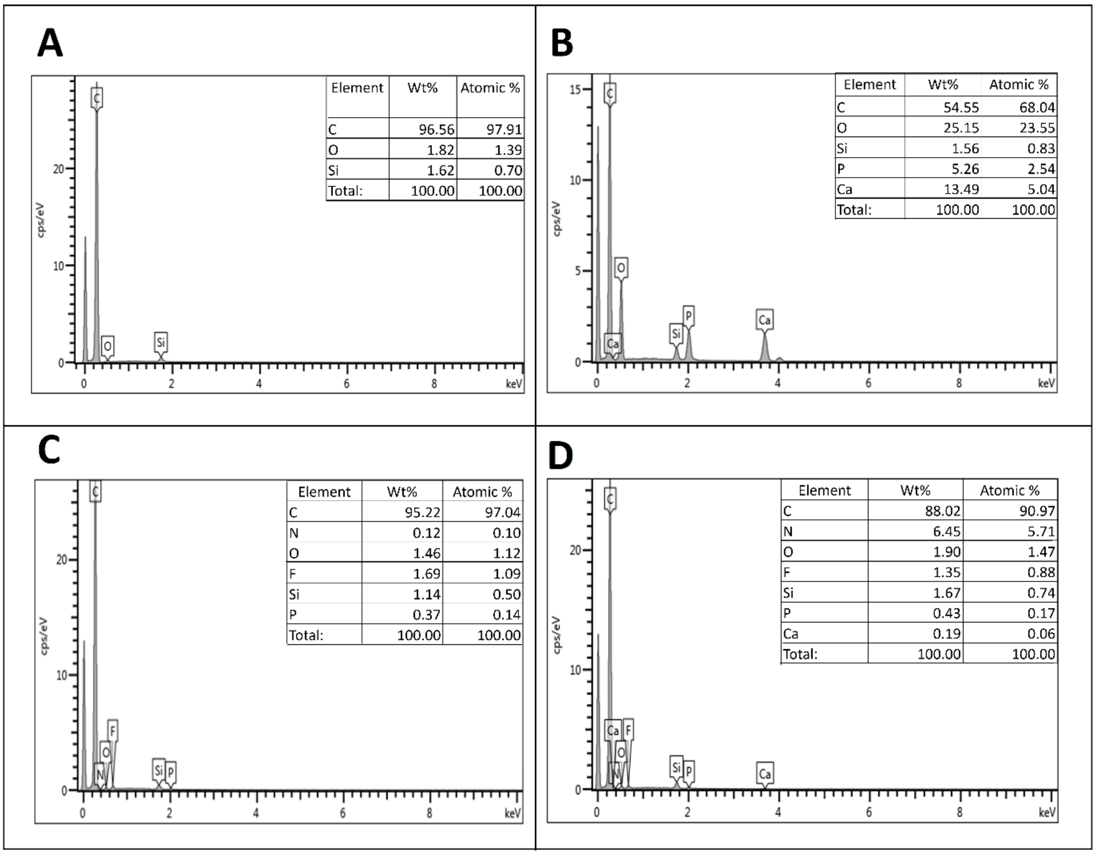

Figure S1. Graphs representing the elemental percentages of (**A**) PGE, (**B**) HaP, (**C**) IL and (**D**) HaP-IL-PGE obtained by EDX analysis.

Table S1. The average anodic peak currents (I_a) with their calculated surface areas (n = 3).

Electrode	I_a (µA)	Surface Area (cm^2)
PGE	87.4 ± 10.3 (RSD % = 11.7 %)	0.27
HaP-PGE	92.2 ± 12.2 (RSD % = 13.21 %)	0.29
IL-PGE	108.7 ± 7 (RSD % = 6.4 %)	0.31
HaP-IL-PGE	115.9 ± 8.7 (RSD % = 7.5 %)	0.35

Table S2. The average R_{ct} values of each of electrodes (n = 3) with the decrease % ratio calculated contrast to the one of PGE.

Electrode	R_{ct} (Ohm)	Decrease % at Rct
PGE	116.5 ± 2.1 (RSD % = 1.8 %)	-
HaP-PGE	54.6 ± 14.5 (RSD % = 26.6 %)	58.2%
IL-PGE	33.2 ± 2.6 (RSD % = 8.1 %)	71.5%
HaP-IL-PGE	30.8 ± 2.4 (RSD % = 7.4 %)	73.5%

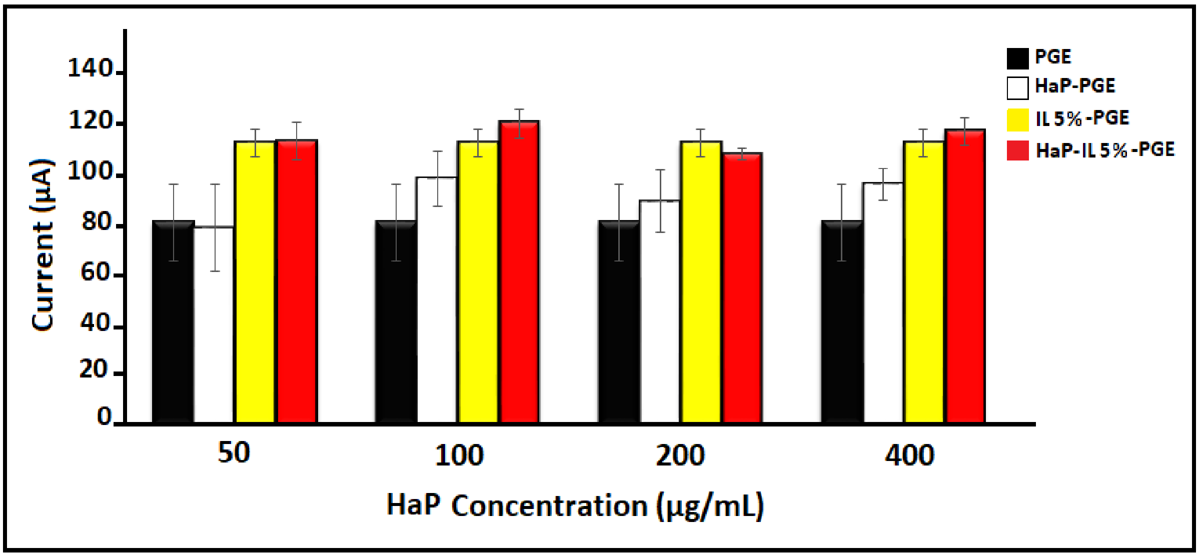

Figure S2. Histograms representing the average oxidation signals measured before and after modification of different HaP concentrations onto the electrode surfaces in the presence of 5 % IL (*n* = 13).

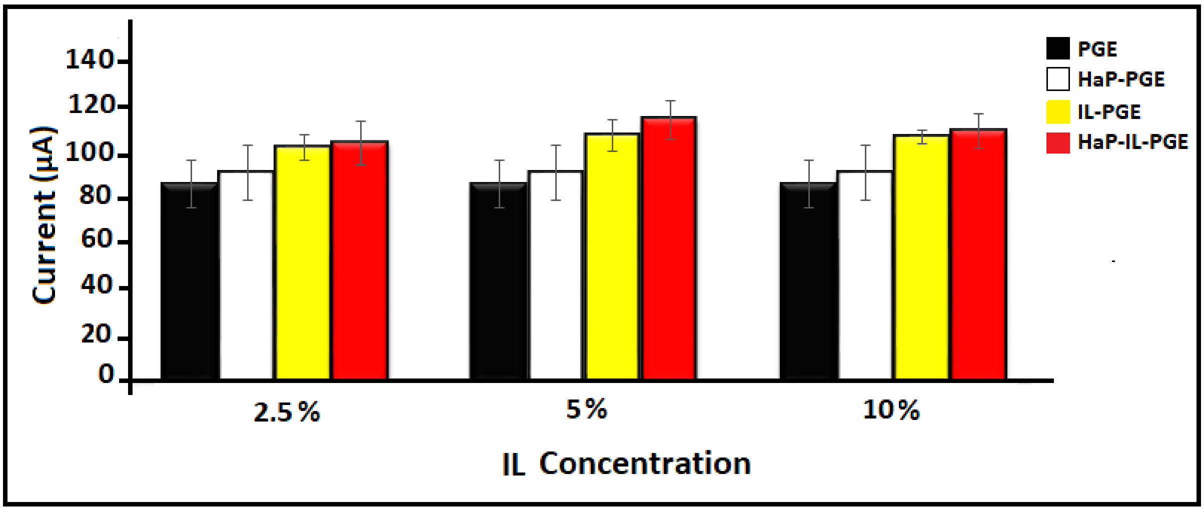

Figure S3. Histograms representing the average oxidation signals measured before and after modification of different IL percentages onto the electrode surfaces in the presence of 100 µg/mL HaP (*n* = 9).

Table S3. The effect of IL % onto the response with the average anodic peak currents (I_a) (*n* = 9).

		**IL %**	**I_a (µA)**
**Electrode**	**IL-PGE**	*2.5 %*	103.8 ± 5.7 (RSD % = 5.5 %)
		5 %	108.7 ± 7.0 (RSD % = 6.4 %)
		10 %	108.2 ± 2.9 (RSD % = 2.7 %)
	**HaP-IL-PGE**	*2.5 %*	105.6 ± 9.4 (RSD % = 8.9 %)
		5 %	115.9 ± 8.7 (RSD % = 7.5%)
		10 %	110.9 ± 7.5 (RSD % = 6.8%)

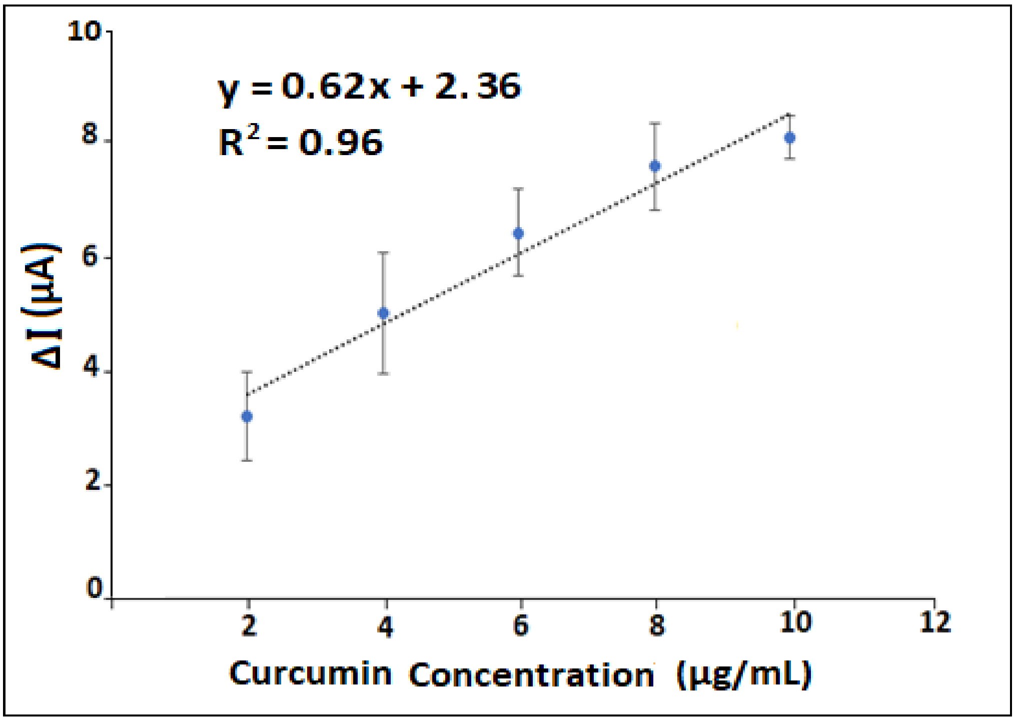

Figure S4. Calibration plot based on the average Curcumin oxidation signals in the presence of various Curcumin concentrations between 2 and 10 μg/mL using HaP-IL-PGEs (*n* = 9).

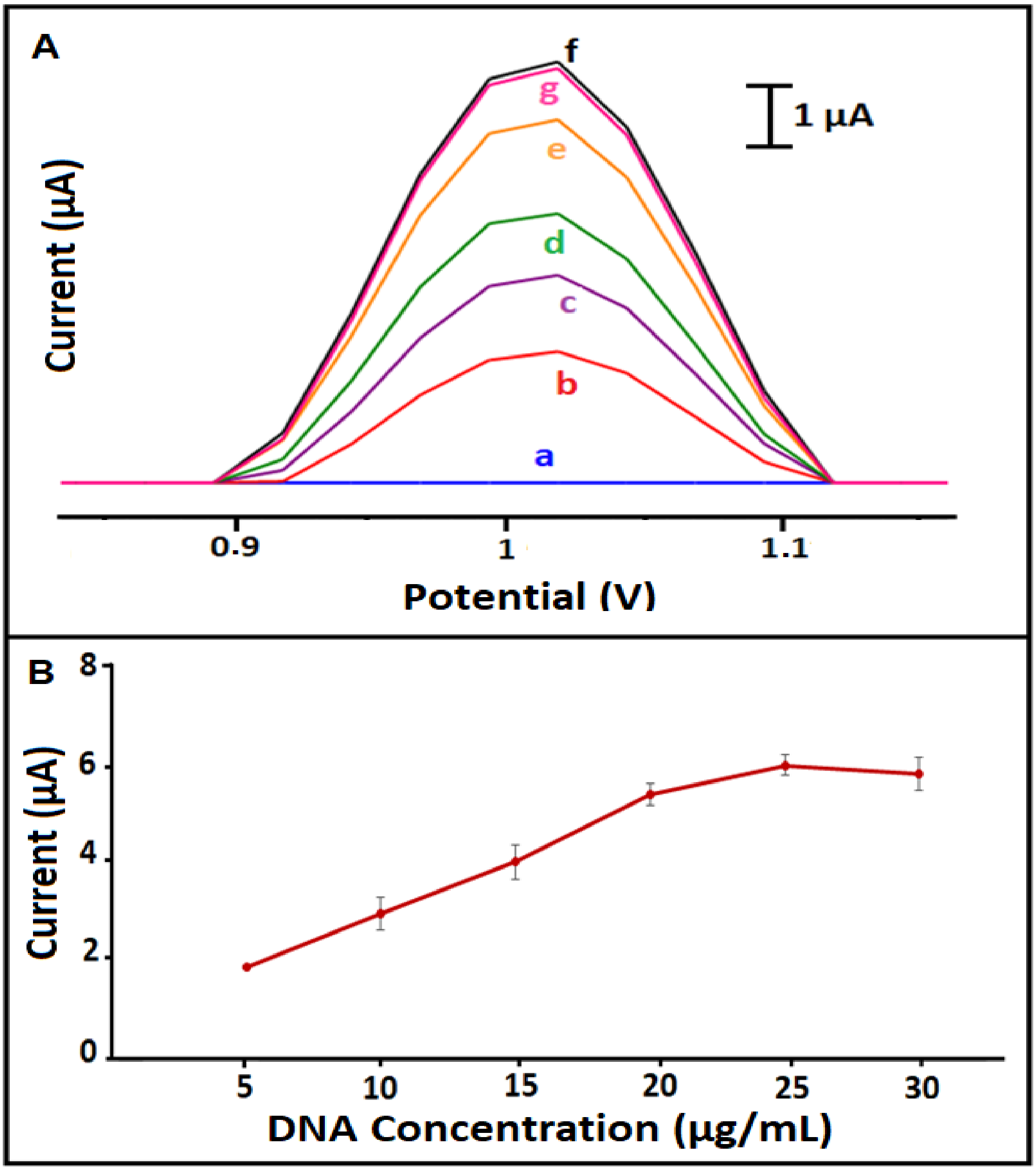

Figure S5. (**A**) DPVs representing the average guanine signals obtained after immobilization of (a) 5, (b) 10, (c) 15, (d) 20, (e) 25, (f) 30 μg/mL ctDNA onto the surface of HaP-IL-PGEs. (**B**) The line graph based on the average guanine oxidation signals obtained by HaP-IL-PGE (n = 3).

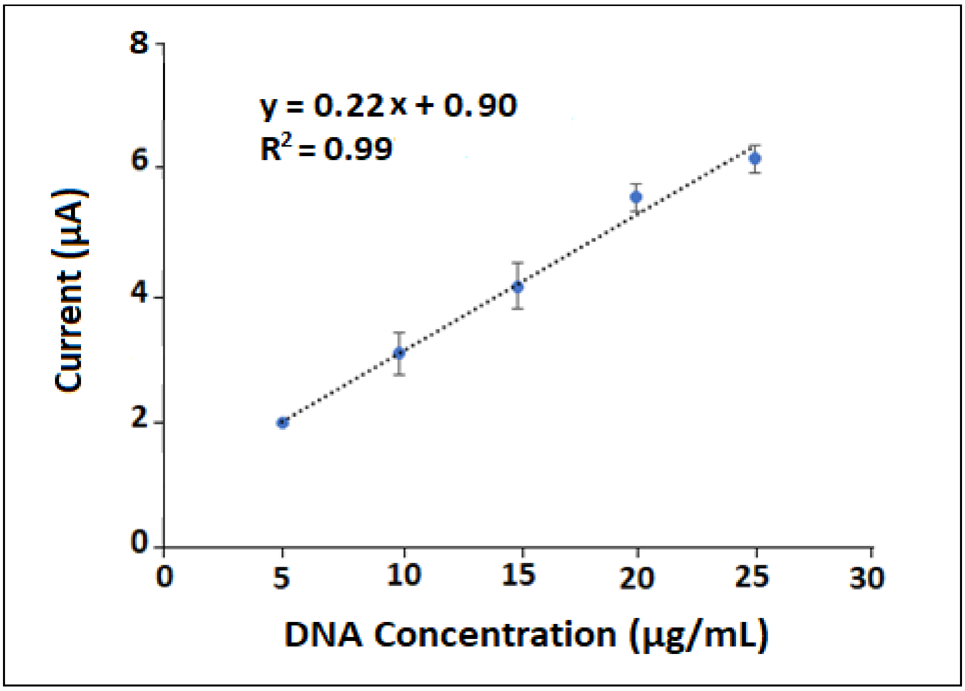

Figure S6. Calibration plot based on the average guanine oxidation signals in the presence of various DNA concentrations between 5 and 25 µg/mL using HaP-IL-PGEs (n = 3).

Table S4. The average oxidation signals of Curcumin before and after different interaction times and change ratios after interaction process between Curcumin and ctDNA (n = 6).

Interaction time (min)	Curcumin signal before interaction (µA)	Current (I, µA) Curcumin signal after interaction (µA)	Change % at Curcumin signal	Curcumin signal before interaction (µA)	Delta Current (ΔI, µA)* Curcumin signal after interaction (µA)	Change % at Curcumin signal
1	5.1 ± 0.6	5.2 ± 1.2	3.2 % ↑	3.4 ± 0.6	3.6 ± 1.2	4.8 % ↑
3	7.3 ± 0.6	6.1 ± 0.7	15.7 % ↓	5.6 ± 0.6	4.5 ± 0.7	20.2 % ↓
5	8.5 ± 1.4	7.7 ± 1.1	9.1 % ↓	6.9 ± 1.4	6.1 ± 1.1	11.3 % ↓

*the average signal of control experiment by HaP-IL-PGE was measured at + 0.56 V as 1.6 ± 0.2 µA (RSD % = 9.63 %, n = 6) that was also overlapping with the oxidation signal of Curcumin measured at + 0.56 V. Therefore ***Delta Current (ΔI)*** was calculated by substracting the control signal of HaP-IL-PGE from curcumin signal.

Table S5. The average oxidation signals of Curcumin before and after different interaction times and decrease ratios after interaction process between Curcumin and PCR samples (n = 6).

Interaction time (min)	Curcumin signal before interaction (µA)	Current (I, µA) Curcumin signal after interaction (µA)	Decrease % at Curcumin signal	Curcumin signal before interaction (µA)	Delta Current (ΔI, µA)* Curcumin signal after interaction (µA)	Decrease % at Curcumin signal
1	5.5 ± 1.1	5.4 ± 0.4	2.2 %	4 ± 1.1	3.8 ± 0.4	3.1 %
3	8.0 ± 0.8	7.7 ± 0.8	4.7 %	6.5 ± 0.8	6.1 ± 0.8	5.8 %
5	9.3 ± 1.3	9 ± 0.6	3.6 %	7.8 ± 1.3	7.4 ± 0.6	4.3 %

*the average signal of control experiment by HaP-IL-PGE was measured at + 0.56 V as 1.6 ± 0.2 µA (RSD % = 9.63 %, n = 6) that was also overlapping with the oxidation signal of Curcumin measured at + 0.56 V. Therefore ***Delta Current (ΔI)*** was calculated by substracting the control signal of HaP-IL-PGE from curcumin signal.

Table S6. The average oxidation signals of guanine before and after different interaction times and change ratios after interaction process between Curcumin and PCR samples (n = 6).

Interaction time (min)	Guanine signal before interaction (µA)	Guanine signal after interaction (µA)	Decrease % at Guanine signal
**1**	4.6 ± 0.3 (RSD % = 7 %)	3.8 ± 0.7 (RSD % = 16.99 %)	16 %
**3**		3.2 ± 0.2 (RSD % = 4.3 %)	30 %
**5**		2.6 ± 0.3 (RSD % = 13.5 %)	44.1 %
